# The classification of intracranial aneurysm neck: a single center research experience

**DOI:** 10.1186/s41016-018-0138-3

**Published:** 2018-12-06

**Authors:** Cai-Qiang Huang, De-Zhi Kang, Liang-Hong Yu, Shu-Fa Zheng, Pei-Sen Yao, Yuan-Xiang Lin, Zhang-Ya Lin

**Affiliations:** Department of Neurosurgery, The First Affiliated Hospital, Fujian Medical University, No.20 Changzhou Road,Taijiang District, Fuzhou, 350004 Fujian Province China

**Keywords:** Intracranial aneurysms, Microsurgery clipping, Aneurysm neck classification, Aneurysm neck curved surface

## Abstract

**Background:**

There is associating with incidence of unfavorable outcomes compared to microsurgical clippings. We are in order to investigate the outcomes of microsurgical clipping for intracranial aneurysms and determine the ideal clipping methods for different aneurysm subtypes.

**Method:**

Retrospectively analyzed the clinical characteristics and follow-up data (completely recorded) of 123 patients with 128 aneurysms were treated. 20 cases were treated as control group from October 2013 to December 2013. Since January 2014, aneurysms were classified base on the 20 cases of aneurysm imaging data. 103 patients were treated as experimental group, the classification of aneurysms previously proposed was used to estimate the way of surgery, and the guiding value of the genotype was verified according to the intraoperative findings. The proposed aneurysm classification is based on the virtual surface of the aneurysm and the parent artery, the aneurysm neck was classified as follows: subtype I, the curved surface of the neck is a single curved surface; subtype II, the neck is hyperboloid; subtype III, neck is a three-curved surface. Aneurysms were divided into further subtypes according to the ratio of the width of the aneurysm neck surface and the length of the artery circumference: subtype A, the ratio of the aneurysm neck surface to the parent artery was not more than 0.5; subtype B, more than 0.5. There are some clamping methods include simple, sliding, interlocking and hybrid.

**Results:**

In the control group, patients did not undergo a suitable clipping scheme without classification of aneurysm neck (unclassed clipping). While causing the occurrence of occlusion adverse events, including neck residual, Tumor artery stenosis, electrophysiological changes, the lack of blood supply and so on. The experimental[page1image12073600]group was analyzed by using a predetermined clipping scheme (classed clipping), and the use of aneurysms clamps was approximately the same as expected. Compared the preoperative assessment with the actual situation, the consistency of the control group was 50% and the experimental group was 96%. Adverse events of classed clipping is 2%, another is 60%. There is a significant difference between the two groups (*P* < 0.05).Classed clipping of subject IA and IB are simple (mean 1.2 and 1.3 clips); classed clipping of subject IIA is simple and interlocking(mean 1.2 clips); classed clipping of subject IIB is sliding and hybrid(mean 2.05 clips); classed clipping of subject IIIA and IIIB are hybrid(mean 2.3 clips).

**Conclusion:**

There is a higher consistency in surgery through the above classification of preoperative assessment of clipping. There was no adverse event of intracranial aneurysm clipping in the clipping mode selected by the above classification, and satisfactory surgical clipping rate was achieved and no recurrence was found.

## Background

Intracranial aneurysm is a common cerebrovascular disease in the neurosurgery, what due to intracranial vascular aneurysm like projections of vascular pathological changes caused by abnormal structure, the rate is 4%–6% [1]. Microsurgical interventions significantly reduced the risk of aneurysmal subarachnoid hemorrhage, severe cerebral vasospasm and brain swelling [2]. Currently, endovascular treatment is accepted by many neurologists [3, 4]. But for the treatment of bifurcation and complex aneurysms, there is still beneficial to the complete clipping of these aneurysms neck surfaces by craniotomy clipping [5, 6]. The space configuration of the aneurysm neck curved surface should be taken into consideration when clipping the neck of the aneurysm [7–10]. The residual neck of aneurysm is one of the most common causes of aneurysm recurrence, residual aneurysm neck is affected by many factors including aneurysm clip selection, placement and combination, resulting in residual and recurrent aneurysms, and controllable factors in most hospital department of neurosurgery physicians only who has clinical experience and surgical skills can provide the equipment support [11, 12]. The value of aneurysmal neck surface has always been ignored in aneurysmal neck types, so this paper made a summary and discussion on 241 patients with intracranial aneurysm for where First Affiliated Hospital of Fujian Medical University has adopted the craniotomy clipping treatment.

## Methods

A retrospective analysis has been made on 241 patients includes 246 aneurysm records and follow-up data in neurosurgery of First Affiliated Hospital of Fujian Medical University from October 2011 to October 2016. The inclusive criterion is diagnosing intracranial aneurysms and treating by using 3D image reconstruction what based on computed tomography angiography (CTA) and digital subtraction angiography (DSA). The exclusion criterion is all of fusiform aneurysms, dissecting aneurysms, blood blister-like aneurysm, and aneurysms with the bypass clipping or wrapping treatment [13].

### General data

This study contains the data of patient quantity, the number of aneurysms, and their clinical data as reflected in age, sex, timing of surgery, Hunt-Hess classification, aneurysmal neck surface types, and clipping methods (Table 1).

**Table 1:** The data of demographic characteristics

Patient and aneurysms	241 Patient, 246aneurysms
average age,year	57
gender(male/female)	117:129
time,d	
< 3	118
> 3	128
Hunt-Hess	
I to III	154
IV toV	92
classification of aneurysms	
Unused	138
Used	108
classification	
class Ia	24
class Ib	24
class IIa	16
class IIb	18
class IIIa	14
class IIIb	12
clipping	
simple clipping	143(85:58)
parallel clipping	49(31:18)
interlocking clipping	36(17:19)
mixed clipping	18(5:13)

The numbers in the parentheses in the right column indicate the use of the pre - and post occlusion of the aneurysm neck

### Imaging data

241 patients were diagnosed intracranial aneurysm by cranial CTA or DSA, who are treated with the microsurgical craniotomy aneurysm clipping according to their aneurysm size, multiple aneurysms, orientation, and location. The responsible vessels will be separated and aneurysm will be clipped in the surgery. (Table 2).

**Table 2:** Imaging data of intracranial aneurysms

Patient and aneurysms	241 Patient, 246 aneurysms
size,mm	
< 5	130
5 to 10	86
10 to 25	30
Multiple aneurysms	5
Aneurysm orientation (left / right)	133:113
Anterior circulation	
Internal carotid artery	26
Anterior choroidal artery	3
Anterior cerebral artery	14
Anterior communicating artery	69
Middle cerebral artery	63
Posterior communicating artery	61
Posterior circulation	
Vertebral artery / base artery	8
Posterior cerebral artery	2

### The types of aneurysmal neck

**Fig. 1:**
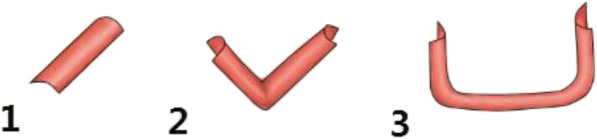
The geometric structure of the neck surface of the aneurysms (I, II, III): 1.1 single surface, 1.2 hyperbolic surface, and 1.3 multi-surface

**Fig. 2:**
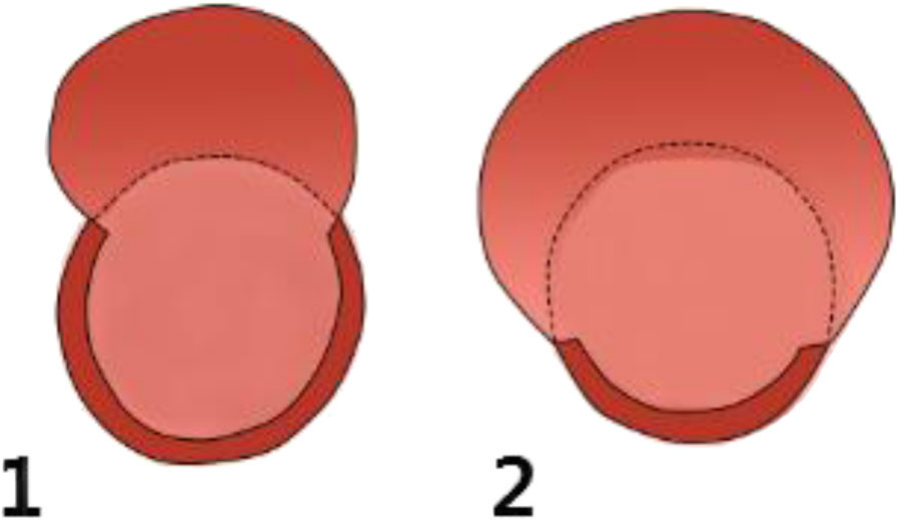
Aneurysm neck surface occupies the size of the perimeter of the responsible vessels (A, B): 2.1 A type (the neck surface is less than the 1/2 of the tumor artery circumference), and the 2.2 B type (the tumor neck surface is smaller than the 1/2 of the tumor artery circumference)

**Fig. 3:**
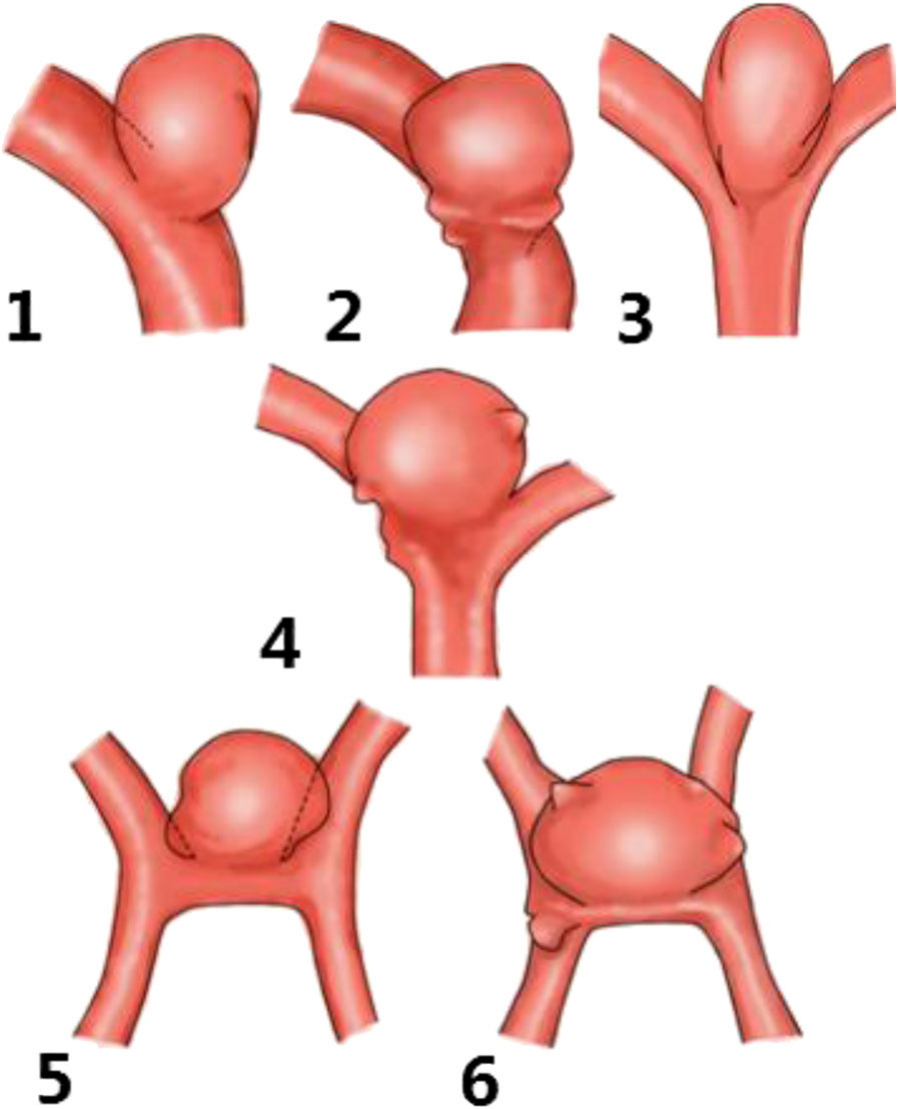
Aneurysm neck type: 3.1 of type A (neck curved single surface, and the surface is less than the parent artery circumference 1/2, 3.2 of type B (neck curved single surface, but greater than the parent artery circumference 1/2), 3.3 A (type II aneurysm neck is double curved surface, and less than the parent artery), 3.4 week long 1/2 II type B (a double neck curved surface, but greater than the parent artery circumference 1/2), 3.5 A (type III neck surface is curved, and less than the parent artery circumference 1/2), 3.6 B (type III aneurysm neck surface a surface, which is larger than the parent artery circumference 1/2)

Type I, II and III (Fig. 1) respectively represent single, double and triple surfaces. A and B subtypes (Fig. 2) respectively represent the surface of aneurysmal neck less than 1/2 of the circumference of the parent artery or long than 1/2 of the circumference of the parent artery. Type I to IIIB are respectively showed in Fig. 3.

### Micrographic clipping surgery

The cerebrospinal fluid was released by opening the relative cistern to reduce the pressure in the cranial cavity. After the relaxation of brain tissue, the operating space.

was exposed to further separate the cistern and lateral fissures, revealing the parent artery and exposing the aneurysmal neck (the situation of blood vessels and the number of blood branch vessels), under direct vision to grasp the status of aneurysm and its adjacent relations. Whether selecting the clipping mode to clip aneurysm is reasonable in preoperative assessment. According to the actual situation to adjust the aneurysm clipping to clip the aneurysmal neck, it needs to completely expose the proximal part of responsible artery to facilitate the temporary blocking of the blood supply of the parent artery (temporary blocking time is limited in 10 min). When monitoring the condition of brain function, it is necessary to quickly clip the aneurysm. After clipping the aneurysmal neck, it needs to check whether there is aneurysmal neck residual, whether there is any nerves are clipped wrongly. As for the vasospasm, it is available to flush by Nimodipine and normal saline to reduce vasospasm. Furthermore, aneurysm clip can get reinforcement with a little fiber cotton and biological glue.

### Clipping methods

It is necessary to contact the aneurysm neck state, intraoperative environment and surgical approach to develop a scientific and appropriate aneurysm clip combination schemes, which includes simple clipping, parallel clipping, interlocking clipping and mixed clipping. Simple clipping is a method that using the single aneurysmal clipping to go along with vessels in order to clip the aneurysm (Fig. 4.1). Parallel clamping means that along the surface of the aneurysm, more than two aneurysm clips clip the aneurysm in a parallel way (Fig. 4.2). Interlocking clipping is using a similar “ring or ear” window-type aneurysm clip combination, which is created by the aneurysm clip and the end of linear aneurysm clip to achieve a complete aneurysmal neck clipping. And make a choice of choosing the double clipping or multiple aneurysmal clipping according to the situation of intracranial aneurysmal neck surface (Fig. 4.3). According to the three methods above, hybrid clipping reshape the original tube wall structure of responsible vessels and branch vessels on the basis of the combination of interlocking clipping and parallel clipping methods (Fig. 4.4).

**Fig. 4:**
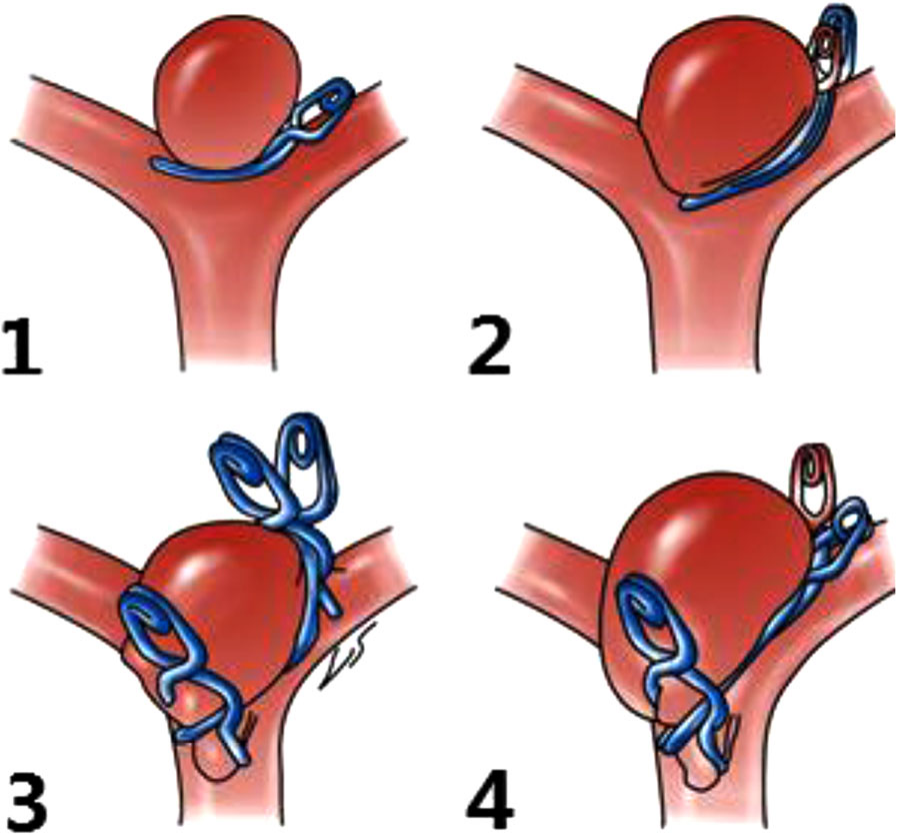
Intracranial aneurysm clipping: 4.1 simple clipping, 4.2 parallel clips, 4.3 interlocking clips, and 4.4 mixed clips

Split - type clipping method proposed by IA type aneurysm is a simple clipping, the clipping methods for IB type is also a simple clipping. But IIA aneurysm clipping is the combination of the simple clipping and interlocking clipping, and IIB is the combination of parallel clipping mixed clipping. IIIA is the mixed clipping, while IIIB type is the mixed clipping based on the parallel clipping and interlocking clipping.

### Statistical analysis

Statistical data should be made a statistical analysis by using SPSS20.0 software. Prognostic factors should be used the logistic regression analysis and linear regression test of single factor and multivariate factors. The *p* < 0.05 error is statistically significant.

## Results

### Clipping methods

Among the 241 patients, through two types of preoperative assessment the surgeon’s experience and aneurysmal neck type, 246 aneurysms have been simulated the aneurysm clipping method. The experiential evaluation is 138 patients which is same with aneurysmal neck type. Changing clipping methods that is consistent with the operation is 68 patients with the concordance rate of 47%; Type assessment that is consistent with the operation is 104 patients, 4 of these have changed the methods according to the actual requirements of the vessels. Therefore, the concordance rate is 96% (Table 3).

**Table 3:** Preoperative assessment of intracranial aneurysms and the actual situation in the operation

	Consistent	Intraoperative changes	Consistency rate	The value of P
Experience	68	70	49%	< 0
Classification	104	4	96%	

### Surgical results

Before type is proposed, the patient of aneurysm was 138. After type is proposed, the patient of aneurysm is 108. Before type is proposed, 125 patients are normal and 13 patients are unmoral after the surgery. However, after type is proposed, 106 patients are normal and 2 patients are unmoral after the surgery (Table 4).

**Table 4:** Monitoring indicators of intracranial aneurysm clipping

Type	Monitoring index						Post closure adverse events
	Error bars	Electrophysiology	TCD	fluorescence	Residual	CT	
Untyped	4/138	5/138	–	–	2/138	2/138	13/138
Typed	0	1/108	–	–	0	1/108	2/108
Total	4/138	6/246	–	–	2/138	3/246	15/246

Adverse events: cause certain damage to the local aneurysm bearing artery and stimulate a series of inflammatory reactions and hemodynamic changes, which may lead to the occurrence of electrophysiology, TCD and residual recurrence

Among the 138 patients with intracranial aneurysms diagnosed before the aneurysmal neck type is proposed, 25 patients have not suffered from the ischemic or postoperative cerebral infarction, and 13 had suffered from permanent or transient ischemic lesions. There were 4 patients are wrongly clipped, 5 patients suffered from electrophysiological changes (3 of them recovery decreased, but 2 of them did not recover), 2 patients suffered from aneurysmal neck residual, 2 patient suffered from computed tomography (CT) infarction after the surgery (1 patient suffered from left frontal lobe parietal infarction, 1 patient suffered from left basal ganglia infarction) (Table 4). Among the 108 patients with intracranial aneurysms who were diagnosed and treated by aneurysmal neck type, according to intraoperative monitoring, 106 patients had been perfect clipped without ischemia and postoperative cerebral infarction. Only two patients have suffered from ischemic situation. One suffered from intraoperative electrophysiological changes (recovery after a transient reduction), the other suffered from cerebral infarction in his postoperative CT review (bilateral temporal lobe medial).

### Demographic data

Using X2 to test various factors, we obtain that single factor analysis shows that aneurysm neck type has a significant difference in the type clipping group and non-type clipping group (*P* < 0.05); While it does not have a significant difference in other 8 factors which includes age, gender, timing of surgery, Hunt-Hess classification, location of aneurysm, aneurysm size and multiple aneurysms (*P* > 0.05) (Table 5).

**Table 5:** Analysis of the clipping factors of intracranial aneurysms

influence factor	cases	normal	adverse event	χ2	P
Age				0.04	> 0.5
≤65	166	158	8		
> 65	80	73	7		
Gender				0.1	> 0.1
male	113	106	7		
female	133	125	8		
Time,d				0.03	> 0.9
< 3	118	111	7		
> 3	128	120	8		
Hunt-Hess				0.004	> 0.95
< IV	154	144	10		
IV to V	92	87	5		
Size,mm				0.008	> 0.99
< 5	130	123	7		
5 to 10	86	80	6		
10 to 25	30	28	2		
aneurysms				0.01	> 0.92
Multiple	5	5	0		
Single	241	226	15		
Orientation				0.01	> 0.99
left	133	125	8		
right	113	106	7		
Classification				6.06	< 0.01
Untyped	138	125	13		
Typed	108	106	2		
Position				3.34	> 0.75
ICA	26	25	1		
ACA	17	16	1		
ACoA	69	65	4		
MAC	63	59	4		
PCoA	61	58	3		
PC	10	10	0		

### Selecting type clipping methods based on the statistical results of aneurysmal neck type

According to the aneurysmal neck type, it is necessary to choose clipping methods according to different statistical trends (*p* < 0.05, Table 6). Type Ia and type Ib aneurysm clipping choose the simple clipping with an average usage of 1.2 and 1.3 aneurysm clips. Aneurysm clipping mode of type IIa is using both simple clipping and interlocking clipping to make a aneurysmal neck reconstruction. In special circumstances, it can use parallel clipping to help complete the huge aneurysm clipping with an average usage of 1.2 aneurysm clips. Type IIb aneurysm clipping ideally uses both parallel clipping and mixed clipping with an average usage of 2.05 aneurysm clips. Type IIIa aneurysm clipping requires to use an average of 2.3 aneurysm clips when clipped, and its ideal state is making use of the mixed clipping. IIIb aneurysm clipping requires to use several aneurysm clips (with an average usage of 2.3 aneurysm clips).

**Table 6:** Different clamping methods based on the selection of aneurysmal neck subtypes

Type	Clamping				adverse changes
	simple	parallel	interlocking	mixed	
Ia	(16/24)	(6/24)	(2/24)	0	
Ib	(17/21)	(1/21)	(3/21)	0	
IIa	(11/15)	(1/15)	(2/15)	(1/15)	1
IIb	(4/19)	(7/19)	(3/19)	(5/19)	1
IIIa	(1/15)	(5/15)	(3/15)	(6/15)	
IIIb	(1/14)	(3/14)	(5/14)	(5/14)	

1. The numbers in parentheses indicate that the number of aneurysms is divided by the subtype to classify the total number of aneurysms

2. Through statistical analysis, the total variation of X - 98.5, V 15, P < 0.005; linear regression - component X 74.8, v 1, *P* < 0.005; deviation from linear regression - component X 23.7, V 14, P 0.025–0.05. The linear regression component and the deviation of linear regression all have statistical significance. It can be considered that there is a correlation between the type of tumor neck and the way of clipping and it is a linear relationship

## Discussion

In regard to the preparation of intracranial aneurysms surgery, fully obtaining aneurysm size and its etiology (fusiform, blisters and thrombus types) and other related information do contribute to the development of aneurysm diagnosis and treatment programs [5, 6]. Based on what is using craniotomy and clamping operation to have a detailed understanding of the relationship of intracranial aneurysm between in the blood vessels in tumor projection and offending vessels on the aneurysm neck surface will help to build the space required for intracranial aneurysm clipping Geometric Information [5, 8, 10]. In this respect, few studies have classified intracranial aneurysms based on their surface features and geometric features. This classification can be used to determine clipping way by evaluating the intracranial aneurysms account for the proportion of the liability artery, and the relationship between the surface of the artery defect and the branch vessel.

The size of the tumor neck of intracranial aneurysms is determined by the part of the parent artery cohesion, which developed and extended by arterial aneurysm. Sometimes, it uses thin-wall and branch vessel to connect with each other. In general, under the light microscope, the features of aneurysmal wall are the destruction of normal elastic fibers [14]. Therefore, it can be achieved the complete closure of intracranial aneurysms through the treatment of this part. In this study, we combine the proportion of offending vessels in aneurysm thin wall (aneurysm neck surface) to classify aneurysmal neck into six subtypes. The significance of this classification is to focus on the actual range of aneurysm neck and the offending vessel surface, which need to reconstruct.

Recalling the different subtypes of clipping method according to different types of aneurysm neck, First, the arterial aneurysm of subtype A and subtype B is a simple clipping method, because their aneurysm neck surface in the same level have a single closed line. Only in the face of a huge B-type aneurysm need to use the chain clip to assist to clip. Second, the type IIB and III aneurysm clipping method is a mixed clipping, the reason is that the aneurysm neck surface has multiple closed lines in different directions (a certain angle constituted by the level of the main vessel and the level of the branch vessel). Therefore, it is need to mix clipping to cure the arterial aneurysm based on the chain or parallel clip. It is impossible for people who blindly hope that by simple clipping method to completely clipping all forms of intracranial aneurysms. Through classifying aneurysm subtype to consider whether the thin wall of aneurysm neck across the offending vessels extends to the reverse side of the blood vessels, resulting to clip difficulties. Furthermore, using multiple aneurysm clips to remodel aneurysm neck surface, so as to keep the blood flowing smoothly. Before clipping the arterial aneurysm along the level of the main vessel, how to clip as much of the rest as possible is the key to make microsurgery decision. In many cases, the surgeon uses multiple aneurysm clips of different shapes to completely closing the intracranial complex aneurysms and fully rebuilding offending vessels [3, 4]. By reconstructing aneurysmal neck, the combination of clipping not only shows the restoration of an anatomical structure of the offending vessel, but also maintains the normal supply of cerebral blood flow.

As the 3D printing aneurysms has proposed by people, it can print out the actual size of similar tumors and vascular to apply in preoperative assessment. The study of this classification system has completely based on the findings of the surgery, rather than just in accordance with the preoperative angiography. In the face of neurological injury events, which easily appears in the process of complicated shape of intracranial aneurysm interlocking, we should use existing monitoring tools to predict the neural functional defect caused by parent artery clipping, so that we can timely adjust the surgical approach to minimize postoperative neurological deficits, scientifically and effectively to avoid neurological damage. In addition, we should have a full understanding of the exact location of arterial aneurysm neck, reducing the neck overlap caused by the process of using permanent aneurysm clip. During the process of permanent interlocking aneurysms, debugging and error adjustment for tumor clipping may be due to the movement of the device to cause damage to the surrounding branches of the aneurysm neck rupture or rupture of the aneurysmal neck and premature rupture of aneurysmal sac [15].

The most important goal in the treatment of intracranial aneurysms is to sustain the integrity and minimal occurrence of complications associated with intracranial aneurysm closure. Some studies report the incidence of microsurgical complications in patients with intracranial aneurysms, which is 2% to 5% [16–18].

In terms of neck aneurysm classification, the reconstruction of aneurysm neck surface has always been ignored. How to better restore the aneurysm neck of the aneurysm neck surface is the pursuit of aneurysm neck surgery. The residue of the aneurysmal neck is one of the most common causes of aneurysm recurrence. The residue of the aneurysmal neck is affected by many factors, including the selection and placement of aneurysm clips, which in turn leads to residual and recurrence of aneurysms. In most of hospitals, the controllable factors are only provided by the neurosurgeons’ clinical experience and surgical skills as well as supporting devices [11, 12]. 3D printing technology apply to intracranial aneurysm treatment. 3D printing model can use the physical model to design the surgical plan, selecting the best surgical method and the appropriate aneurysm clipping. It can also simulates high similarity and personalized design features of the aneurysm [19]; The 3D model facilitates surgical planning, particularly for the design of multiple arterial aneurysm for the preoperative planning and tumor clipping of giant aneurysms, can realize the complex arterial aneurysm with a single custom tumor clipping. In addition, through the fine clamping technique, the selection of tumor clamps and surgical procedures according to the type of tumor neck provides a more safe and feasible treatment strategy for intracranial aneurysms.

There are still some limitations to our current research. First, we excluded the intracranial aneurysm attached to large thrombus, because it has the high risk associated with therapy (the rupture of the interlocking process and the poor stability of the upper clipping). Our goal, however, is to determine a suitable sub-type to manage the clip craniotomy intracranial aneurysm. The treatment of thrombosis of aneurysms should to use the other therapies, such as intravascular thrombectomy and tamponade. Second, the choice of clipping type of intracranial aneurysmal neck type is inevitable to existing different subjective preference of the surgeon. For example, we can use the direct parallel of stacking tumor clip in the process of clipping, not using many tumors clipping to lock the tumor to reconstruct a complex aneurysm with wide neck. Third, because the research design is a retrospective study, this study did not consider other morphological factors except for the geometric size of the neck, which may lead to bias in the results. Therefore, continuous prospective studies are required to include all possible considerations, such as atherosclerosis, multiple mesenchyme and secondary tumor. Fourth, based on the diversity of the aneurysm orientation, this study put aside the influence of the location and orientation, and did not consider the difference of the difficulty of the surgical approach and the difficulty of the closure. However, our results provide evidence that the clipping approach chosen for aneurysm classification based on the classification of aneurysm neck surface is a perfect result for achieving a good surgical outcome without postoperative complications and aneurysm recurrence.

## Conclusion

This study was designed to select the classification of clipping based on the morphological classification of the aneurysm neck surface, using for the treatment of intracranial complex aneurysms. Our study suggested that the treatment of intracranial aneurysms based on the classification of the aneurysm neck surface, to assist microsurgical techniques in clipping the aneurysm, and avoid postoperative complications and aneurysm recurrence.

## Abbreviations

CT: Computed tomography
CTA: Computed tomography angiography
DSA: Digital subtraction angiography
